# The Mutational Landscape of Acute Myeloid Leukemia and Its Impact

**DOI:** 10.3390/ijms27135797

**Published:** 2026-06-26

**Authors:** Tarindhi Ratnayake, Clifford Liongue, Alister C. Ward

**Affiliations:** 1 School of Medicine, Deakin University, Waurn Ponds, Geelong, VIC 3216, Australia; s222416671@deakin.edu.au (T.R.); c.liongue@deakin.edu.au (C.L.); 2 Institute for Mental and Physical Health and Clinical Translation (IMPACT), Deakin University, Waurn Ponds, Geelong, VIC 3216, Australia

**Keywords:** acute myeloid leukemia, mutations

## Abstract

Acute myeloid leukemia (AML) is one of the most common types of hematological malignancies and a leading cause of cancer deaths. It is characterized by the rapid accumulation of typically immature myeloid cells that serve to disrupt the production of mature cells, leading to a range of clinical sequelae. The role of recurrent chromosomal aberrations has long been appreciated in this disease, but a myriad of gene mutations have been increasingly acknowledged as having important roles. This review provides a comprehensive overview of the mutational landscape of AML, discussing the various genetic lesions in terms of their function, classification, etiological role, prognostic value, therapeutic impact, detection, and monitoring, with a particular focus on gene mutations.

## 1. Introduction

Acute myeloid leukemia (AML) is an umbrella term for a heterogeneous group of hematological malignancies characterized by uncontrolled proliferation of typically immature myeloid cells with concomitant disruption of normal hematopoiesis [1]. AML can arise *de novo* or secondary to other hematological malignancies like myelodysplastic syndrome (MDS) and myeloproliferative neoplasms (MPNs) [2]. AML is a common disease, comprising around 35% of adult cancers and 15–20% childhood cancers, with poor outcomes, as reflected in an overall survival rate of 30% [3].

Recurrent chromosomal abnormalities have been recognized in AML patients for many decades, being important in disease etiology and prognosis. However, almost one-half of adult and a quarter of pediatric patients possess a normal karyotype. More recently, large-scale sequencing approaches have identified a plethora of gene mutations in AML patients that impact disease presentation, course, prognosis, and their response to specific treatments [4].

This review aims to provide a comprehensive picture of the mutational landscape of AML, detailing the different genetic lesions identified and their contribution to leukemogenesis, including how they cooperate in this process, with a focus on gene mutations. Such insight is essential for facilitating the development of more effective diagnostic, prognostic, and therapeutic approaches for this disease.

## 2. Categories of Genetic Lesions in AML

**Figure 1:**
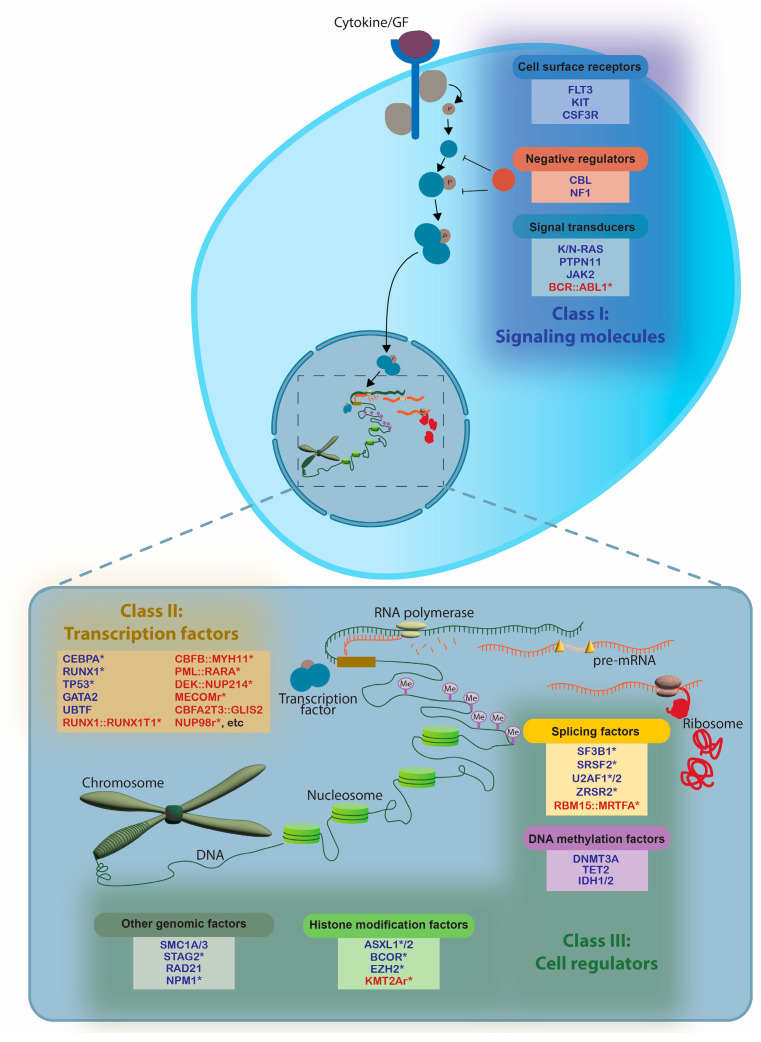
Mutational landscape of acute myeloid leukemia. Schematic of the most common mutations found in acute myeloid leukemia grouped by their principal functional class (and sub-class), positioned according to their major site of action within a cell, of which selected components are shown, including a close-up of nuclear elements. Gene mutations are shown in blue, while chromosomal aberrations are in red, with those representing defining lesions of AML categories indicated with an asterisk and rearrangements designated as ‘r’.

A two-hit model for AML was first proposed nearly a quarter of a century ago [5]. In this model, two different classes of genetic lesions were required, with those in so-called ‘class I’ genes providing a proliferation and/or survival advantage and those in ‘class II’ genes instead disrupting differentiation. This model has provided a useful framework but has increasingly failed to capture the complexity underpinning the molecular etiology of AML. With the increasing emphasis on specific genetic rather than phenotypic changes in disease classification and management, it is timely to recast and expand this model to accommodate the totality of lesions observed. In this revised framework, class I lesions are better defined as those that augment cell signaling—typically gain-of-function (GOF) mutations in cell surface receptors and downstream signal transducers or loss-of-function (LOF) mutations in their negative regulators—that serve to positively impact proliferation and/or survival. Class II lesions instead impact key transcription factors, being mainly LOF mutations, which result in defective differentiation. A third ‘class III’ grouping represents lesions in a range of molecules involved in the regulation of critical cellular processes, including epigenetic regulators such as those involved in DNA methylation and histone modification, other genome factors, and splicing factors. These mutations can be GOF or LOF, often with broader impacts, including augmenting mutations in the other classes [6,7,8]. This framework provides a useful scaffold to understand the roles of gene mutations observed in AML (Figure 1).

## 3. Gene Mutations in AML

### 3.1. Class I: Signaling Molecules

#### 3.1.1. Cell Surface Receptors

FMS-like receptor tyrosine kinase 3 (FLT3) is a receptor tyrosine kinase (RTK) expressed on early hematopoietic stem cells (HSCs), B-cell progenitors, myeloid progenitors, monocytes, and dendritic cells. FLT3 initiates the PI3K/AKT and RAS/RAF/MEK/ERK signaling pathways to affect the survival, maturation, and proliferation [9]. Mutations of the *FLT3* gene are one of the most common genetic lesions in AML, being found in 30% of cases. The majority involve internal tandem duplications (ITDs) in the juxtamembrane domain that trigger ligand-independent activation of FLT3, leading to enhanced proliferation of hematopoietic cells, although activating point mutations in the tyrosine kinase domain (TKD) have also been observed [9].

KIT is another RTK expressed on HSCs, mast cells, and various non-hematopoietic cells. KIT signals via the PI3K/AKT, RAS/RAF/MEK/ERK, and JAK/STAT pathways to regulate self-renewal and differentiation of HSCs [10,11]. KIT mutations have been reported in about 25% of AML cases. These mutations are mostly localized to the juxtamembrane and TKD sequences, resulting in ligand-independent activation [11].

Colony-stimulating factor 3 receptor (CSF3R) is a homodimeric cytokine receptor expressed on various hematopoietic cells, particularly neutrophils and their precursors. CSF3R signals via the JAK/STAT, RAS/RAF/MEK/ERK, and PI3K/AKT pathways, mediating the proliferation of myeloid precursors and differentiation and survival of neutrophils, as well as HSC mobilization [12]. Various *CSF3R* mutations have been identified in myeloid neoplasms, with an incidence of 2–4% in AML. The majority of these are point mutations localized to the transmembrane domain of the receptor, leading to ligand-independent activation or truncating cytoplasmic domain mutations, resulting in enhanced signaling [13], with the latter especially common in patients with preceding severe congenital neutropenia (SCN) [14].

#### 3.1.2. Signal Transducers

The RAS proteins comprise a family of small GTPases expressed in almost all types of cells, typically bound to the cytosolic region of the plasma membrane. These proteins act downstream of multiple cytokine and growth factor receptors, activating the RAS/RAF/MEK/ERK and PI3K/AKT cascades that regulate the proliferation of various hematopoietic cells [15]. Amongst the three *RAS* genes, *NRAS* and *KRAS* are important in AML, with *NRAS* mutations accounting for about 10–15% of AML cases and *KRAS* mutations accounting for 5–10% of AML cases [16].

Protein-tyrosine phosphatase nonreceptor type 11 (PTPN11) is widely expressed in hematopoietic cells and other diverse tissues, including the heart and kidney. This protein is part of the signal transduction of many cytokines and growth factors, contributing to the activation of many downstream signaling pathways like the JAK/STAT, RAS/RAF/MEK/ERK, and PI3K/AKT cascades [17]. PTPN11 positively regulates hematopoiesis, affecting the renewal and mobilization of HSCs, and is also involved in lymphopoiesis [18]. Mutations in *PTPN11* are found in about 4% of AML cases [19]. These mutations are mostly identified in the N-terminal SH2 and PTP domains and result in hyperactivation of the protein [18].

Janus kinase (JAK2) is a member of a family of tyrosine kinases that facilitate intracellular signaling downstream of a variety of cytokines and other receptors with which they associate, notably including STATs. These in turn regulate key cellular processes by affecting the transcription of genes involved in the proliferation, differentiation, and function of their target cells, with JAK2 being critical for definitive erythropoiesis. *JAK2* mutations are most commonly found in the TKD or the regulatory pseudokinase domain, resulting in hyperactivation in each case [20]. Such GOF mutations of JAK2 have been observed in about 6% of de novo AML cases, but they are also common secondary to several MPNs [21,22].

#### 3.1.3. Negative Regulators

Casitas B-lineage lymphoma (CBL) protein is an E3 ubiquitin ligase enzyme ubiquitously expressed in hematopoietic cells. These proteins direct ubiquitin to specific target proteins, thereby facilitating their degradation [23]. This typically negatively regulates the signaling downstream of RTKs principally via the PI3K/AKT and RAS/RAF/MEK/ERK cascades, thereby impacting the proliferation and survival of hematopoietic cells [24]. Mutations of *CBL* are found in about 1–2% of AML cases and are usually LOF mutations impacting the ring finger region of the protein, leading to increased proliferation [25].

Neurofibromatosis type 1 (NF1) neurofibromin is a GTPase-activating protein (GAP) expressed in all cell types. It acts as a negative regulator for activated RAS proteins and plays a critical role in regulating hematopoietic cell proliferation and differentiation [26]. *NF1* mutations have been reported in about 5% of AML [27]. These LOF mutations are localized to the GAP-related domain and downregulate the downstream RAS/RAF/MEK/ERK pathway [28].

### 3.2. Class II: Transcription Factors

CCAAT/enhancer binding protein-α (CEBPA) is a member of a family of transcription factors primarily expressed in myeloid cells, with highest expression in cells of the granulocytic, monocytic, and eosinophilic lineages. CEBPA is a critical mediator of myelopoiesis, regulating the maturation of myeloid cells by inducing the expression of key genes such as *CSF3R*, *IL6R*, and *CEBPE* [29]. *CEBPA* mutations are reported exclusively in AML and have been identified in about 15% of patients [29]. These LOF mutations are primarily categorized into two major groups: mutations in the N-terminal domain that result in overexpression of truncated versions of the protein and in-frame deletions in the C-terminal bZIP domain that impact DNA binding and often involve both alleles [30,31].

Runt-related transcription factor 1 (RUNX1) is a transcription factor that is expressed in both myeloid and lymphoid progenitors. This protein acts as an essential regulator of early hematopoiesis and affects the proliferation, differentiation, and survival of HSCs [32]. It operates by interacting with the core-binding factor beta (CBFB) to mediate complex regulation of transcription of many genes related to hematopoiesis [33]. The *RUNX1* gene is one of the most frequently targeted genes in acute leukemia, with LOF point mutations identified in about 10% of AML cases [34].

Tumor protein 53 (TP53) is a transcription factor regulating both the cell cycle arrest and DNA repair pathways [35]. Under normal conditions, it is expressed at low levels in all tissues but is activated by cellular stresses, particularly DNA damage, where it arrests the cell cycle and triggers apoptosis. It plays an integral role in hematopoiesis by regulating the proliferation of bone marrow mesenchymal cells and helps maintain the population of progenitor cells [36]. LOF mutations in the *TP53* gene have been reported in 5–10% of AML patients, typically being missense mutations affecting the DNA-binding domain [37].

GATA2 is a member of the GATA family of tissue-specific transcriptional regulators that play a crucial role in hematopoiesis. GATA2 is expressed in HSCs and progenitor cells and plays a major role in erythroid/ megakaryocytic differentiation [38]. LOF mutations in the *GATA2* gene are found in about 5% of AML cases and are usually localized to the N-terminal zinc finger domain, affecting DNA binding and repressing transcriptional activity [39].

The upstream binding transcription factor (UBTF) is a ubiquitously expressed member of the high-mobility group (HMG) box protein family [40]. UBTF is a key regulator of ribosomal RNA (rRNA) gene transcription and facilitates the recruitment of RNA polymerase I to transcriptionally active regions of chromatin within the nucleolus [41]. *UBTF* mutations are recurrent genetic alterations in AML, occurring in approximately 4% of pediatric cases and 3% of adult patients [40,42]. These mutations are predominantly GOF in-frame tandem duplications within exon 13 that affect the HMG-box 4 domain of the protein [42]. About 30% of UBTF-mutated AMLs are secondary to hematological malignancies such as MDS [41].

### 3.3. Class III: Cell Regulators

#### 3.3.1. DNA Methylation Factors

DNA methyltransferase 3A (DNMT3A) is a highly conserved member of the DNA methyltransferase family that is highly expressed in HSCs [43]. This enzyme controls the methylation of genomic DNA and regulates the expression of genes that facilitate the proliferation and differentiation of HSCs [43,44]. LOF mutations in the *DNMT3A* gene are found in about 20% of AML cases, being higher in adult than pediatric patients [45]. These mutations usually occur within the methyltransferase domain (MTD) of the protein, ablating its activity and serving to increase self-renewal capacity and decrease lineage differentiation [46].

Ten-eleven-translocation 2 (TET2) is part of a DNA dioxygenase family that promotes DNA demethylation, being highly expressed in HSCs and granulocytes. TET2 has been shown to play a vital role in maintaining normal hematopoiesis by regulating the lineage differentiation of HSCs, particularly monocytes [47]. LOF mutations in *TET2* are frequently observed in AML, including around ~27% of all patients, particularly in those with prior MDS [48]. These mutations are spread throughout the gene, spanning from exon 3 to 11 and often the C-terminal catalytic domain, resulting in a loss of enzymatic activity, and causing target genes to remain methylated [49].

The isocitrate dehydrogenases (IDHs) are widely expressed metabolic enzymes that are involved in oxidative respiration. IDH1 is restricted to the cytoplasm/peroxisome of the cells, while IDH2 is found in the mitochondria. Both are involved in the conversion of isocitrate to α-ketoglutarate, which is an essential cofactor for certain histone and DNA demethylases [50]. Mutations in the *IDH1* and *IDH2* genes are primarily found in AML, with the most common being missense mutations IDH1-R132 and IDH2-R140 reported in about 12% and 20% of AML patients, respectively [51]. These GOF mutations result in altered activity such that, instead of producing α-ketoglutarate, they produce 2-hydroxyglurate. The loss of α-ketoglutarate leads to hypermethylation of histone and DNA, leading to altered gene expression and decreased erythroid differentiation [52].

#### 3.3.2. Histone Modification Factors

Additional sex combs-like 1 and 2 (ASXL1 and ASXL2) are non-catalytic components of the polycomb repressive complex (PRC) expressed in almost all tissues [52]. The PRC is involved in histone deubiquitination, which impacts chromatin assembly to facilitate the expression of genes responsible for stem cell functions [53]. In particular, ASXL1 is involved in the proliferation and differentiation of myeloid progenitor cells, whereas ASXL2 is associated with self-renewal and lineage differentiation of HSC [54,55]. *ASXL* genes are collectively amongst the most frequently mutated epigenetic regulators in AML. LOF *ASXL1* mutations are found in about 5–17% of AML, and LOF *ASXL2* mutations are found in about 23% of AML cases [56].

BCL6 co-repressor (BCOR) is a transcriptional co-repressor ubiquitously expressed in human tissues. This protein facilitates the formation of PRC2, mediating histone ubiquitination that serves to repress the transcription of genes promoting the differentiation of hematopoietic progenitor cells toward the myeloid lineage. In addition, BCOR also regulates the development of lymphocytes and impacts mesenchymal stem cell function [57,58]. LOF mutations that span the length of the *BCOR* gene have been reported in about 4% of AML with normal karyotypes and 8% of secondary AML cases associated with MDS [59].

Enhancer of zeste homolog 2 (EZH2) is a histone methyltransferase that is a core component of PRC2. This protein is expressed in proliferating hematopoietic cells and influences the balance between the proliferation and differentiation of HSCs by promoting transcriptional repression [60]. LOF mutations in the *EZH2* gene resulting in loss of histone methylation have been reported in about 4% of AML cases. The majority of these mutations are reported in the C-terminal domain, which is responsible for methyltransferase activity [61].

#### 3.3.3. Other Genomic Factors

Cohesin is a multimeric protein structure that comprises four core subunits: structural maintenance of chromosome protein 1A (SMC1A) and 3 (SMC3), stromal antigen 2 (STAG2), and RAD21. These collectively form a ring-shaped structure around the DNA double helix that regulates DNA looping, which is important in maintaining chromatin structure [7]. Each of the cohesion components is ubiquitously expressed, with a key role in regulating the balance between proliferation in HSCs and progenitor cells [62,63,64]. LOF mutations in the *SMC1A* and *SMC3* genes have an incidence of 2–5% in AML [65,66], being generally missense mutations that affect the hinge domain and alter the chromosome binding of the encoded proteins [62]. LOF *STAG2* mutations are also prominent in AML, with an incidence of 2–12% [66], and distributed across the protein [67]. Similarly, LOF mutations in *RAD21* affecting all domains of the protein are found in AML with an incidence of 3% [68].

Nucleophosmin 1 (NPM1) is a histone chaperone protein that participates in nucleosome formation by interacting with both the core H2A/H2B/H3 histones and the H1 linker histone. This is important for maintaining the stability of active and repressed regions of the genome to facilitate appropriate gene expression [69]. NPM1 is highly expressed in proliferating cells, including HSCs, and functions as a key regulator of hematopoiesis, affecting the proliferation of progenitor cells [70]. GOF mutations in the *NPM1* gene are found in about 20–30% of AML cases [71]. These mutations are mostly localized at exon 12 of the gene to generate an alternative nuclear export signal domain, causing the NPM1 protein to accumulate in the cytoplasm instead of its normal nuclear location, thereby disrupting its normal functions but also mediating new functions [72].

#### 3.3.4. Splicing Factors

Splicing factor 3B subunit 1 (SF3B1) is a subunit of the spliceosome 3b complex, which is a core component of spliceosomes that regulates RNA splicing in various cells, including myeloid progenitors. SF3B1 is particularly important in heme biosynthesis, mitochondrial metabolism, and the NF-kB pathway that regulates lineage differentiation of erythroid precursors [73]. LOF mutations in the *SF3B1* gene have been detected in about 3% of AML cases, where they mostly occur as heterozygous point mutations of the C-terminal HEAT domain to impact splicing [74,75].

Serine and arginine-rich splicing factor 2 (SRSF2) is a pre-RNA splicing factor protein that is expressed in various cell types, including hematopoietic cells. SRSF2 is essential for genomic stability and lineage differentiation of hematopoietic cells [76]. Mutations in the *SRSF2* gene have been identified in AML at a relatively high frequency (6–10%) [8,77] and are generally GOF mutations that alter the specificity of RNA binding and disrupt the splicing of mRNAs encoding hematopoietic regulators [75].

U2 small nuclear RNA auxiliary factors 1 and 2 (U2AF1 and U2AF2) are two spliceosome components widely expressed in human cells, including hematopoietic cells. These proteins are vital in maintaining the survival and function of hematopoietic progenitor cells, particularly those in the erythroid lineage [78]. LOF mutations in *U2AF1* are detected in about 4% of AML cases, whereas those in *U2AF2* mutations are rarer (<1%) [8,77].

ZRSR2 is a splicing factor that recognizes 3’ intron splice sites by interacting with other components of the pre-spliceosome assembly, including the U2AF1/U2AF2 heterodimer and SRSF2. This protein regulates myeloid differentiation, particularly the erythroid lineage, by preventing mis-splicing [79]. LOF mutations in *ZRSR2* are very diverse, including nonsense, frameshift, missense, and splice-site mutations, collectively identified in just less than 5% of AML cases [8,77].

## 4. Genetic Lesions in AML Classification

The most recent AML classification systems use genetic lesions as the main criteria for defining specific sub-categories [80,81] (Table 1). These include an array of chromosomal abnormalities, which can also be placed in the proposed three-class functional framework (Figure 1). From this, it is clear that the majority of these affect class II genes, specifically translocations and/or inversions affecting *RUNX1*, *CBFB*, *RARA, DEK*, *MECOM*, *NUP98*, etc., but also including translocations affecting *KMT2A* and *RMB15*, which impact class III genes, and a sole class I gene, the *BCR::ABL1* translocation. Importantly, the new classification systems also incorporate gene mutations. These principally impact class III genes (*NPM1*, *ASXL1*, *BCOR*, *EZH2*, *SF3B1*, *SRSF2*, *U2AF1*, and *ZRSR2*), along with several class II genes (*CEBPA*, *RUNX1*, and *TP53*) [80,81].

However, the development of neoplasia involves multiple genetic lesions that act in concert to fully manifest the disease [82]. This includes co-occurring gene mutations that are not part of the classification systems but are common and of clinical importance in AML. With respect to AML categories defined by chromosomal abnormalities, the co-occurring mutations are typically class I. Of these, *FLT3* mutations are the most prevalent, occurring with *PML::RARA*, *DEK::NUP214*, and *NUP98* rearrangements, while *KRAS* and/or *NRAS* mutations are the most common with *CBFB::MYH11,* as well as *KMT2A* and *MECOM* rearrangements, and *KIT* mutations are the most prevalent with *RUNX1::RUNX1T1*, being the second most prevalent for *CBFB::MYH11*. Amongst defined gene mutation categories, *DNMT3A* and *FLT3* mutations are most commonly associated with *NPM1* mutations, *GATA2* mutations with *CEBPA* mutations, and rarer chromosomal aberrations with *TP53* mutations [83,84].

## 5. Genetic Lesions in AML Etiology

**Figure 2:**
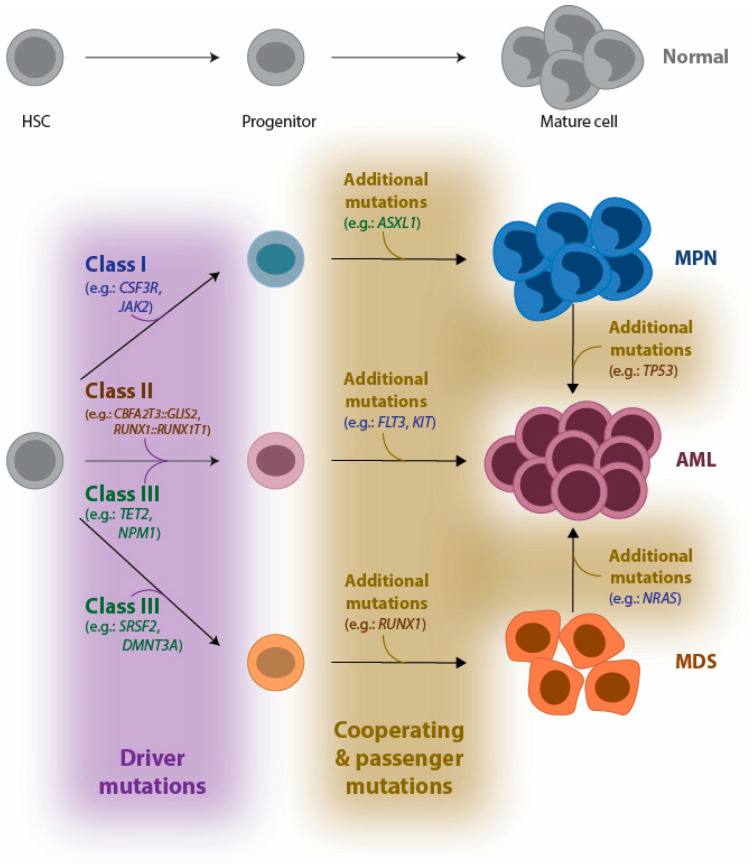
Hematopoiesis and its disruption in primary and secondary AML. The upper panel shows the differentiation of HSC to normal blood cells in a healthy individual, while the lower panel demonstrates how this is disrupted in primary AML (middle) or AML secondary to either MPN (above) or MDS (below). The timing of driver and cooperating/passenger mutations is shown, along with the prevalent gene class for the driver mutations, with examples. Genes—dark blue: class I; brown: class II; green: class III. Cells—grey: normal cells; blue: mutated but phenotypically normal cells in MPN; pink/purple: mutated blast-like cells in AML; orange/brown: mutated dysplastic cells in MDS.

The various genetic lesions identified in AML are important in disease etiology. The early lesions that trigger the onset of neoplasia are often classified as ‘driver’ mutations, which can be identified based on their timing, functional impact, and frequency [85,86]. The numerous other ‘non-driver’ mutations that collaborate with driver mutations to facilitate the progression of the neoplasm are referred to as ‘cooperating’ mutations, while those that are simply coincidental and not involved in the neoplastic process are called ‘passenger’ mutations [82,86,87] (Figure 2).

In AML, the driver genetic lesions are almost exclusively in class II (often chromosomal aberrations) or class III (typically gene mutations). In AML, however, class I mutations are typically cooperating mutations, occurring later and often at high frequency, with the ability to cooperate with class II and III drivers [88]. Their cooperation with class II genes is often very specific, reflecting relevant transcriptional networks, such as *KIT* mutations with *RUNX1* and *CBFB* chromosomal abnormalities [89] and *CSF3R* mutations with *CEBPA* mutations [30]. Cooperation with class III genes is typically broader. For example, *FLT3* mutations often co-exist with *NPM1* mutations, resulting in a significantly worse prognosis [90], but they are also commonly found with mutations in DNA methylation genes [43], while multiple class I genes cooperate with *TET2* [49]. There is also evidence of cooperation between class I genes, especially *FLT3*, *KIT*, and RAS pathway members [11], but there is also evidence of mutual exclusivity, such as between different members of the RAS pathway [16]. Cooperation between gene mutations is also evident for the other classes. Thus, CEBPA not only cooperates with fellow class II members RUNX1 and GATA2 but also ASXL1 and NPM1 from class III [91,92]. There is strong cooperation between a group of class II and III mutations in the MDS-related AML grouping, while within class III, *NPM1* mutations cooperate with those in DNA methylation factors, particularly *DNMT3A*, which is co-mutated in the majority of cases. Mutual exclusivity is also evident, such as between *TET2* and *IDH1/2* mutations [49] and between different cohesion complex members [65].

It is informative to compare this to MPN and MDS, especially since these strongly predispose to AML. In MPN, specific class I genetic lesions represent the key drivers and are present at much higher frequency than in AML, with mutations of *CSF3R* in ~90% of chronic neutrophilic neutropenia (CNL) [13]; mutations of *JAK2* in ~100% of polycythemia vera (PV) and 50–60% of essential thrombocythemia (ET) and idiopathic myelofibrosis (IMF) [93]; mutations of *KIT* in >80% of mastocytosis [94]; and mutations of a common ‘RAS’ pathway comprising *NRAS*, *KRAS*, *CBL*, *NF1*, and *PTPN11* in 85–90% of juvenile myelomonocytic leukemia (JMML) [95], and the chromosomal aberration *BCR-ABL1* in ~100% of chronic myelogenous leukemia (CML) [96]. Such lineage-specific impacts are reflected in the AML sub-types that these mutations are observed in: *CSF3R* mutations in M2, *JAK2* mutations in M5 and M7, *KIT* mutations being highly represented in M2 and M4, and RAS pathway mutations in M4 and M5 subtypes. In MDS, however, the driver mutations are almost exclusively class III, with splicing (e.g., *SRFS2*) and DNA-modifying factors (e.g., *DNMT3A*) predominating [97]. In both diseases, additional mutations are commonly found in genes from the other classes.

## 6. Genetic Lesions in AML Prognosis

The various genetic lesions underpinning AML are increasingly being used as prognostic indicators. These can be categorized from favorable (e.g., *RUNX1::RUNX1T1*) to intermediate (e.g., *FLT3*) to adverse (e.g., *EZH2*) (Table 2).

However, the use of genetic lesions as prognostic indicators is not straightforward, with the constellation of genetic lesions being important. For example, *NPM1* mutations are considered favorable, but only in the absence of *FLT3* mutations, which shifts them to the intermediate category. Such classifications remain incomplete, with the relative risk of most class I and many key class III mutations unclear. They are also being constantly reevaluated; for example, recent work has suggested that *SRSF2* and *STAG2* mutations might be best considered intermediate rather than adverse [98]. Finally, the ELN 2022 classification system presented below was based on young adults receiving intensive therapy, with alternatives subsequently proposed for adult AML patients receiving less-intensive therapy [99] and for pediatric AML patients [100].

## 7. Gene Mutations Informing AML Therapy

The increasing understanding of recurrent gene mutations in AML has not only provided important insights into disease biology but also driven the development of mutation-specific therapeutic strategies (Table 3). Some of these directly target the mutated protein. For example, tyrosine kinase inhibitors have proven effective in AML in which *FLT3* is mutated. First-generation inhibitors, including sorafenib and midostaurin, facilitated improved response rates and survival outcomes when combined with chemotherapy activity, although off-target toxicities were evident largely due to the broad spectrum of tyrosine kinases they could inhibit [101]. These have been supplanted by more selective and potent but less toxic second-generation inhibitors, such as quizartinib, which is approved for monotherapy and in combination with chemotherapy [102], crenolanib [103], and gilteritinib, showing promise against relapsed/refractory AML [104]. IDH inhibitors have also been developed, with ivosidenib and enasidenib now representing important therapies in AML carrying relevant IDH1 or IDH2 mutations, respectively [105,106]. In contrast, one approach for TP53 therapy is to reactivate mutant forms, such as rezatapopt, which targets the Y220C mutant [107].

Targeted approaches are not always effective. For example, FLT3 inhibitors achieve durable responses in only one-third of AML patients carrying *FLT3* mutations, with remission rates ranging from 30 to 40% [114]. This can be due to primary resistance mediated by factors within the bone marrow microenvironment or secondary resistance through acquired on-target *FLT3* mutations or off-target mutations in bypass pathways such as RAS/MAPK, PI3K/AKT, and JAK/STAT. To overcome these mechanisms, next-generation FLT3 inhibitors with activity against resistant mutations have been developed, along with rational combination approaches targeting bypass pathways or the protective bone marrow niche [114,115]. In addition, TP53-targeted approaches have shown limited efficacy and considerable toxicity. Here, immunotherapeutic treatment modalities have emerged as potential strategies to improve outcomes in this high-risk patient population. These include TIM-3 blockade (sabatolimab), immune checkpoint inhibitors, CD123- and CD33-directed bispecific antibodies, and chimeric antigen receptor (CAR)-T or CAR-NK cell therapies [116,117].

Indeed, many of the therapies for specific gene mutations act indirectly. Thus, hypomethylating agents, including azacitidine and decitabine, have demonstrated therapeutic benefit in patients with *IDH1/2* and other mutations through impacts on epigenetic control, particularly when used in combination-based regimens [108]. Similarly, therapies for *NPM1*-mutated AML target various mediators. These include Selinexor, a first-generation inhibitor of XPO1 that facilitates nuclear export of mutant NPM1 [109], along with second-generation eltanexor that addresses some of the toxicity and dosing-related challenges relating to selinexor [69]. Additional therapeutic approaches under investigation for *NPM1*-mutated AML include inhibitors of downstream effector proteins, including revumenib for menin, which is now in phase II/III trials [110], pinometostat for DOT1L H3K79me [111], and WM-3835 for HBO1 H3K14me [46]. Several agents for the treatment of AML harboring splicing factor mutations act to cause synthetic lethality, including RBM39 degraders such as indisulam [112] and inhibitors of the essential spliceosome assembly protein PRMT, such as PRT543 [113].

Individual gene mutations are also prognostic for the effectiveness of other therapies. For example, AML patients with *NPM1*, *IDH1/2*, or splicing factor mutations are typically sensitive to the selective BCL-2 inhibitor venetoclax, whereas those with *TP53* or *FLT3* mutations are frequently resistant [118]. Meanwhile, those carrying *RUNX1* or *CEBPA* mutations are typically more responsive to hypomethylating agents [119].

## 8. Detecting and Monitoring Genetic Lesions in AML

Given the growing clinical importance of genetic lesions, it is important to consider how they are identified and quantified. Histology and immunophenotyping through multiparameter flow cytometry (MFC) remain critical for initial diagnosis, with the underlying genetic lesions subsequently identified by a number of methods [120]. This includes conventional cytogenetics to elucidate chromosomal changes, including many common translocations, supplemented with fluorescence in situ hybridization (FISH) to provide additional sensitivity and accuracy. This is followed by molecular testing for gene mutations used to define disease or risk categories or underpin specific treatment strategies. This is typically achieved using quantitative PCR (qPCR) and specific gene panels, although next-generation sequencing (NGS) approaches offer the widest coverage [120].

Quantification of the remaining leukemic cell burden following treatment, or minimal residual disease (MRD), is critical for assessing treatment efficacy, ongoing disease monitoring, and informing treatment decisions. Several approaches are suitable for this purpose, each with unique strengths and weaknesses. This includes MFC using leukemia-associated phenotypes, which is widely available in hospital settings, being relatively quick, low cost, and broadly applicable across different AML patients—although with a lower sensitivity (10^−3^–10^−4^) [121]. MRD analysis using qPCR has similar availability and time/cost effectiveness but offers higher sensitivity (10^−4^–10^−6^), albeit with reduced applicability [122]. NGS is emerging as an alternative approach despite modest sensitivity (10^−3^–10^−4^) and high cost and turnaround time due to its wide applicability and ability to follow clonal evolution [122]. Specific MRD assays have been recommended according to risk group and mutational status, with thresholds for positive, low-level positive, and negative MRD burden categories to underpin appropriate qualitative responses [123].

## 9. Conclusions

AML is an aggressive cancer with poor clinical outcomes. Understanding of this disease has developed from insights based on recurrent chromosomal abnormalities largely impacting proliferation, survival, and differentiation to a more nuanced understanding where a network of genetic lesions—including a myriad of gene mutations—collectively disrupt hematopoiesis. This includes ‘driver’ mutations that trigger the onset of this disease and ‘cooperating’ mutations affecting disease development, prognosis, and treatment outcomes.

This review provides a useful framework to understand the mutational landscape of AML and its impacts. However, doing so inevitably necessitates simplification. Firstly, of the myriad gene mutations reported in AML, there has been a focus on a subset of the more frequently mutated genes, especially those used for classification and prognostic or therapeutic purposes. Secondly, not all genetic lesions fit neatly into the different classes, while the GOF/LOF dichotomy fails to fully capture mutations that introduce new functionalities, such as *IDH1/2* [56]. Finally, AML is heterogeneous, with differences between pediatric and adult patients, as well as primary, secondary, and inherited forms. In adult AML, the most common early lesions are mutations in the DNA methylation factors *DNMT3A*, *TET2*, and *IDH2*; the histone modification factor *ASXL1*; and the splicing factors *SRSF2* and *NPM1*—all class III. In pediatric AML, the early lesions instead typically represent chromosomal abnormalities mainly impacting class II genes, including those impacting *RUNX1* and *CBFB* and also the class II histone modification factor *KMT2A* [84,88]. Therapy-related forms show a preponderance of chromosomal aberrations impacting chromosomes 5 and/or 7, or leading to complex karyotypes, and *TP53* mutations following chemotherapy, and translocations involving *KMT2A*, *RUNX1*, and *RARA* following topoisomerase II inhibitors [124]. Germline predispositions include several of the genes discussed already, such as the class II *CEBPA*, *TP53*, *RUNX1,* and *GATA2* and the class I RAS pathway, but they also involve many others, including *DDX41*, *ANKRD26*, *ELANE*, and *FANC* [125]. Therefore, it is important that clinical responses are nuanced to take these differences into account.

Improved knowledge of the gamut of genetic lesions in AML has significantly increased our understanding of disease biology and contributed to new systems of classification and prognosis and the development of precision-based therapeutic approaches. However, disease heterogeneity, clonal evolution, and the development of resistance to therapy remain major clinical challenges, highlighting the need for continued efforts to explore mutation-specific and combination treatment strategies.

## Figures and Tables

**Table 1:** Specific genetic lesions are used in AML classification.

Genetic Lesion	WHO 5th Ed	ICC
*BCR::ABL1*	+	+
*RUNX1::RUNXT1*	+	+ ^1^
*CBFB::MYH11*	+	+
*PML::RARA*	+	+ ^2^
*DEK::NUP214*	+	+
*MECOM* rearrangement	+	+ ^3^
*NUP98* rearrangement	+	+ ^4^
*KMT2A* rearrangement	+	+ ^5^
*NPM1::MLF1*	+	+
*KAT6A::CREBBP*	+	+
*MNX1::ETV6*	+	+
*FUS::ERG*	+	+
*CBFA2T3::GLIS2*	+	+
*RBM15::MRTFA*	+	−
*PRDM16::RPNI*	−	+
*PICALM::MLLT10*	−	+
*NPM1* mutation	+	+
*CEBPA* mutation	+	+ ^6^
*TP53* mutation	−	+
MDS-related mutations (*ASXL1*, *BCOR*, *EZH2*, *SF3B1*, *SRSF2*, *STAG2*, *USAF1*, *ZRSR2*)	+	+ ^7^

^1^ A separate category exists for *RUNX1::CBFA2T3*. ^2^ A separate category exists for other *RARA* rearrangements. ^3^ A separate category exists for *GATA2::MECOM*. ^4^ Separate categories exist for *NUP::NSD1* and *NUP::KMD5A*. ^5^ A separate category exists for *MLLT3::KMT2A*. ^6^ In-frame pZIP mutations. ^7^ Includes *RUNX1.*

**Table 2:** Risk classification of specific genetic lesions in AML (ELN 2022 recommendations).

Favorable	Intermediate	Adverse
*CEBPA* ^1^ *NPM1* ^2^ *RUNX1::RUNX1T1* *CBFB::MYH11* *PML::RARA*	*FLT3* *MLLT3::KMT2A*	*ASXL1* *BCOR* *EZH2* *SF3B1* *SRSF2* *STAG2* *U2AF1* *ZRSR2* *RUNX1* *TP53* *DEK::NUP214* *BCR::ABL1* *KAT6A::CREBBP* *MECOM* rearrangement ^3^ *KMT2A* rearrangement Other chromosomal ^4^
	**Unclear**	
*KIT* *CSF3R* *JAK2* *CBL* *NF1* *K/NRAS* *PTPN11*	*GATA2* *UBTF* *NPM1::MLF1* *KAT6A::CREBBP* *MNX1::ETV6* *FUS::ERG* *CBFA2T3::GLIS2* *RBM15::MRTFA* *PRDM16::RPNI* *PICALM::MLLT10* *NUP98* rearrangement	*DNMT3A* *TET2* *IDH1/2* *ASXL2* *U2AF2* *SMC1A/3* *RAD21*

^1^ In-frame bZIP. ^2^ In the absence of *FLT3* mutation. ^3^ Including *GATA2::MECOM*. ^4^ Including −5/del 5q; −7; −17/abn(17p); complex karyotypes, monosomy.

**Table 3:** Therapeutic agents for specific gene mutations found in AML.

Mutation(s)	Mode of Action	Agents	References
*FLT3*	FLT3 inhibitor	sorafenib, midostaurin, quizartinib, crenolanib, gilteritinib	[101,102,103,104]
*IDH1*	IDH1 inhibitor	ivosidenib	[106]
*IDH2*	IDH2 inhibitor	enasidenib	[105]
*TP53*	TP53 reactivator	rezatapopt	[107]
*IDH1/2*	Hypomethylater	azacytidine, decitabine	[108]
*NPM1*	XPO1 inhibitor	selinexor; eltanexor	[69,109]
	Menin inhibitor	revumenib	[110]
	DOTL1 inhibitor	pinometostat	[111]
	HBO1 inhibitor	WM-3835	[46]
Splicing factors	RBM39 degrader	indisulam	[112]
	PRMT inhibitor	PRT543	[113]

## Data Availability

No new data were created in this study.

## Abbreviations

The following abbreviations are used in this manuscript: AML: acute myeloid leukemia; ASXL1/2: additional sex combs-like 1/2; BCOR: BCL6 co-repressor; CAR: chimeric antigen receptor; CBL: Casitas B-lineage lymphoma; CBFB: core binding factor-β; CEBPA: CCAAT/enhancer binding protein-α; CML: chronic myelogenous leukemia; CNL: chronic neutrophilic leukemia; CSF3R: colony-stimulating factor 3 receptor; DNMT: DNA methyltransferase; ET: essential thrombocythemia; EZH2: enhancer of Zeste homolog 2; FISH: fluorescence in situ hybridization; FLT3: FMS-like receptor tyrosine kinase; GAP: GTPase activating protein; GOF: gain of function; HSC: hematopoietic stem cell; IMF: idiopathic myelofibrosis; ITD: internal tandem duplication; IDH: isocitrate dehydrogenase; JAK2: Janus kinase 2; JMML: juvenile myelomonocytic leukemia; LOF: loss of function; MDS: myelodysplastic syndrome; MFC: multiparameter flow cytometry; MPN: myeloproliferative neoplasm; MRD: minimal residual disease; NF1: neurofibromatosis type 1; NGS: next-generation sequencing; NPM1: Nucleophosmin1; PTPN11: protein-tyrosine phosphatase nonreceptor type 11; PV: polycythemia vera; qPCR: quantitative polymerase chain reaction; RTK: receptor tyrosine kinase; RUNX1: runt-related transcription factor 1; SCN: severe congenital neutropenia; SF3B1: splicing factor 3b subunit 1; SMC1A/3: structural maintenance of chromosomes protein 1A/3, SRSF2: serine and arginine rich splicing factor 2; STAG: TET2: ten-eleven translocation 2; TP53: tumor suppressor protein 53; TKD: tyrosine kinase domain; U2AF1/2: U2 small nuclear RNA auxiliary factor ½; UBTF: upstream binding transcription factor.
